# Methylome, transcriptome, and PPARγ cistrome analyses reveal two epigenetic transitions in fat cells

**DOI:** 10.4161/epi.29856

**Published:** 2014-07-10

**Authors:** Hitomi Takada, Yutaka Saito, Toutai Mituyama, Zong Wei, Eiji Yoshihara, Sandra Jacinto, Michael Downes, Ronald M Evans, Yasuyuki S Kida

**Affiliations:** 1 Research Center for Stem Cell Engineering; National Institute of Advanced Industrial Science and Technology (AIST); Tsukuba, Japan; 2 Computational Biology Research Center; National Institute of Advanced Industrial Science and Technology (AIST); Tokyo, Japan; 3 Japan Science and Technology Agency; Kawaguchi, Japan; 4 Gene Expression Laboratory; Salk Institute for Biological Studies; La Jolla, CA USA; 5 Howard Hughes Medical Institute; Salk Institute for Biological Studies; La Jolla, CA USA

**Keywords:** epigenetics, DNA methylation, fat differentiation, reprogramming, adipose derived stem cells, induced pluripotent stem cells, fat cells

## Abstract

Although DNA modification is adaptive to extrinsic demands, little is known about epigenetic alterations associated with adipose differentiation and reprogramming. We systematically characterized the global trends of our methylome and transcriptome data with reported PPARγ cistrome data. Our analysis revealed that DNA methylation was altered between induced pluripotent stem cells (iPSCs) and adipose derived stem cells (ADSCs). Surprisingly, DNA methylation was not obviously changed in differentiation from ADSCs to mature fat cells (FatCs). This indicates that epigenetic predetermination of the adipogenic fate is almost established prior to substantial expression of the lineage. Furthermore, the majority of the PPARγ cistrome corresponded to the pre-set methylation profile between ADSCs and FatCs. In contrast to the pre-set model, we found that a subset of PPARγ-binding sites for late-expressing genes such as *Adiponectin* and *Adiponectin receptor2* were differentially methylated independently of the early program. Thus, these analyses identify two types of epigenetic mechanisms that distinguish the pre-set cell fate and later stages of adipose differentiation.

## Introduction

Adipose tissue is one of the most sensitive and important physiological regulators. In response to physiological changes, such as nutrition intake, calorie restriction, or exercise, adipose tissue controls homeostatic energy balances as a lipid storage or burning organ.^1^^,^^2^ An imbalance between energy intake and expenditure increases fat mass, promotes adipocyte differentiation,^3^ and alters the secretion of adipokines,^4^ eventually leading to obesity. Thus, elucidating the mechanisms underlying epigenetic control of adipogenesis will contribute to the fundamental understanding of metabolic disorders including obesity, one of the major health problems in recent decades.

Adipogenesis is controlled by a complex gene network that converts fibroblast-like adipose precursor cells into lipid-laden adipocytes. In vitro models of adipogenesis using 3T3-L1 cells or harvested human adipose stem cells have revealed a cascade of transcription factors, among which peroxisome proliferator-activated receptor gamma (PPARγ) and CCAAT/enhancer-binding proteins (C/EBPs) function as master regulators.^5^ Recent studies have shown that the transcription of adipogenic genes is accompanied by epigenetic modifications such as histone methylation and acetylation,^6^^-^^8^ indicating that chromatin remodeling has important roles in adipogenesis. However, the complex relationships among DNA methylation, histone modification, and the recognition of transcription factor binding are not fully understood.

DNA methylation, an epigenetic modification, is known as a regulator of gene expression in cell proliferation, differentiation, and reprogramming.^9^^,^^10^ In general, CpG methylation was thought to silence mRNA transcription by altering the accessibility of DNA to transcriptional regulators.^11^ However, recent whole genome MethylC-Seq studies have shown that the roles of DNA methylation in gene regulation vary depending on the genomic context, such as CpG density, or genomic structures, such as promoters and gene bodies.^10^^,^^12^ These data demonstrate that the global trends of DNA methylation and gene expression are complicated. Furthermore, the link between DNA methylation and adipogenic gene expression is not yet understood.

Closer inspection of DNA methylation at specific transcription factor-binding sites would also advance the understanding of the correlation between DNA methylation and gene regulation. Previous studies have shown that the methylation state of transcription factor binding sites impacts the binding ability of transcription factors,^13^ thereby regulating the transcription of the associated genes.^14^ In the process of adipocyte differentiation, PPARγ-binding sites are associated with an active chromatin configuration marked by H3K4me and H3K27ac.^7^^,^^15^ However, the DNA methylation states of PPARγ-binding sites have not been elucidated.

In this study, we analyzed the DNA methylome in adipose-derived stem cells (ADSCs), mature adipocytes (FatCs) differentiated from ADSCs, and induced pluripotent stem cells (iPSCs) reprogrammed from ADSCs to explore DNA methylation dynamics, gene expression, and transcription factor binding during adipogenesis. Our group and Sun et al. recently reported the efficient epigenetic reprogramming of ADSCs to iPSCs^16^^,^^17^ and the global trends of DNA methylation in human embryonic stem cells and iPSC lines^18^; thus, iPSCs, ADSCs, and FatCs are ideal resources for analyzing the DNA methylome without the need to consider the genetic background variations, including SNPs. Here, we present the unexpected result that the methylation state of promoters and gene bodies was not markedly changed upon adipocyte differentiation, whereas a change in methylation state was evident upon reprogramming. Furthermore, the majority of PPARγ cistrome is not obviously changed from ADSCs to FatCs. In contrast, a subset of PPARγ−binding sites is differentially methylated during adipogenesis. These results reveal two types of epigenetic mechanisms during adipogenesis.

## Results

### DNA methylation status in ADSCs, reprogrammed iPSCs, and differentiated FatCs

To explore the epigenetic underpinnings of fat reprogramming and differentiation, we evaluated the DNA methylomes of adipose-derived stem cells (ADSCs), induced pluripotent stem cells reprogrammed from ADSCs (iPSCs), and mature adipocytes differentiated from ADSCs (FatCs). We also profiled the mRNA transcriptome of each cell type to analyze the relationship between methylation status and gene expression. In our protocol, a pure population of FatCs, not contaminated with undifferentiated cells, was carefully collected from floating cells after centrifugation (see Materials and Methods). This procedure yielded reproducible and high-quality data, in which we confirmed strong expression of fat-specific genes (e.g., FABP4) in FatCs (Table S1), but not in ADSCs or iPSCs.

Recent studies revealed that highly expressed genes have low promoter methylation and high gene-body methylation.^10^ Therefore, we first analyzed each cell type separately by comparing the expression of genes whose methylation was low or high in promoters or gene bodies. To investigate the effect of CpG islands, promoters were divided into two classes based on their CpG contents (Fig. 1A), where high CpG promoters contain CpG island-like sequences. Gene bodies were also divided into two classes based on CpG contents, although their discrimination was not clear (Fig. 1C).

**Figure:**
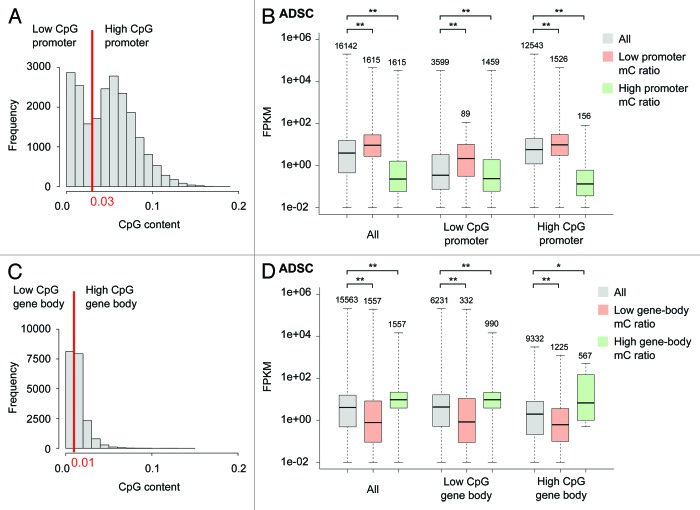
**Figure 1.** Relationship between promoter or gene-body methylation and gene expression in ADSCs. (**A**) Promoter CpG content in the human genome. Low- and high-CpG promoters were divided by the threshold of 0.03. (**B**) The FPKM distributions are shown for all genes, as well as those in the lowest 10% with respect to mC ratio, and those in the highest 10%. *P* values were calculated by the Wilcoxon rank sum test. (**C**) Gene-body CpG content in the human genome. (**D**) Analysis similar to that described for (**B**) was performed for the mC ratios calculated from gene bodies. Symbols *: *P* < 0.05; **: *P* < 10^−3^. Box: 25–75th percentile. The number of plotted genes is shown above each whisker. Genes with no expression in all the three cell types were excluded from the analysis.

In ADSCs, genes with higher promoter methylation showed lower expression (Fig. 1B), whereas this relationship was reversed for gene body methylation; genes with higher gene-body methylation showed higher expression (Fig. 1D). The same tendencies were also seen in iPSCs and FatCs (Fig. S1), even when the two CpG content classes were separately analyzed. Thus, we observed that promoters and gene bodies exhibited opposite methylation-expression relationships in ADSCs, iPSCs, and FatCs. These results extend our knowledge of the methylome-transcriptome relationship to a wider range of cell types.

### Differential DNA methylation during reprogramming and differentiation

We next analyzed the differential methylation of paired cell types (ADSC/iPSC and ADSC/FatC) to assess the changes corresponding to the reprogramming of ADSCs to iPSCs and the differentiation of ADSCs to FatCs. As in the previous section, the effects of the genomic context on methylation dynamics were also considered.

The methylation difference was more drastic in the reprogramming to iPSCs than in the differentiation to FatCs. For reprogramming to iPSCs, hypermethylation (i.e., stronger methylation in iPSCs than in ADSCs) was widespread in promoters (Fig. 2A) and gene bodies (Fig. 2C), and hypomethylation also occurred in a number of regions. In contrast, differential methylation was not evident in promoters (Fig. 2B) or gene bodies (Fig. 2D) during differentiation to FatCs. We identified genes with significantly different levels of methylation by comparing the mC ratios of promoters with those of random genomic loci (see Materials and Methods). Compared with ADSCs, we identified 819 hypomethylated and 291 hypermethylated genes in the reprogramming to iPSCs, whereas only 110 hypomethylated and 73 hypermethylated genes were observed in the differentiation to FatCs. Surprisingly, the stable methylation observed for the differentiation to FatCs did not correlate with gene expression. Despite the relatively subtle differentiation-related differences in methylation among promoters and gene bodies, the gene expression variation between ADCSs and FatCs was similar to that between ADSCs and iPSCs (Fig. 2E and F). These data indicate that the methylation profile of ADSCs is predetermined toward the FatC lineage prior to adipogenic gene expression.

**Figure:**
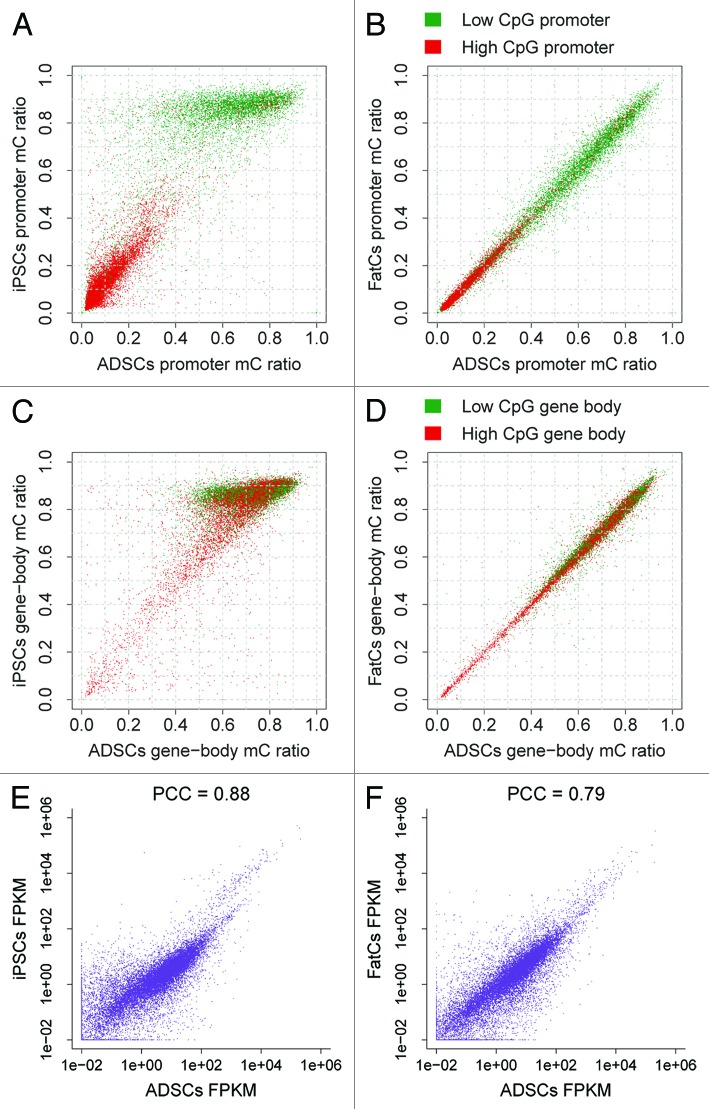
**Figure 2.** Differential methylation between ADSCs, iPSCs, and FatCs. (**A,B**) Differential promoter methylation between ADSCs and iPSCs (**A**) and between ADSCs and FatCs (**B**). (**C,D**) Differential gene-body methylation between ADSCs and iPSCs (**C**) and between ADSCs and FatCs (**D**). (**E, F**) Differential expression between ADSCs and iPSCs (**E**) and between ADSCs and FatCs (**F**). The FPKM variation was evaluated by the Pearson correlation coefficient (PCC). Despite a lack of change in promoter and gene-body methylation, the differentiation to FatCs involves a large variation in expression, as does reprogramming to iPSCs.

In reprogramming to iPSCs, differential promoter methylation showed distinct patterns depending on the CpG content. Low-CpG promoters were highly methylated and were responsible for most of the differential methylation, whereas high-CpG promoters were relatively unchanged and had low levels of methylation (Fig. 2A). Notably, such patterns were prominent in promoter methylation in reprogramming to iPSCs, and were not clearly observed in gene body methylation (Fig. 2C and D).

### Differential promoter methylation is correlated with differential gene expression during reprogramming

Numerous studies have documented that variations in gene expression are accompanied by alterations in DNA methylation.^9^^,^^19^ The large number of genes determined to be differentially methylated in reprogramming to iPSCs motivated us to investigate whether differential methylation correlated with differential expression.

The differential promoter methylation in reprogramming correlated well with differential gene expression. To visualize the correlation, we calculated the enrichment of differentially expressed genes in the plot area of mC ratios (Fig. 3). When all genes were plotted together, hypomethylation and hypermethylation were correlated with activation and repression, respectively (Fig. 3A). We validated this observation by testing the enrichment of differentially expressed genes among differentially methylated genes (Fig. S2), where 248 out of 819 hypomethylated genes were activated, and 174 out of 291 hypermethylated genes were repressed (Fisher exact test, *P* < 0.05 in both cases).

**Figure:**
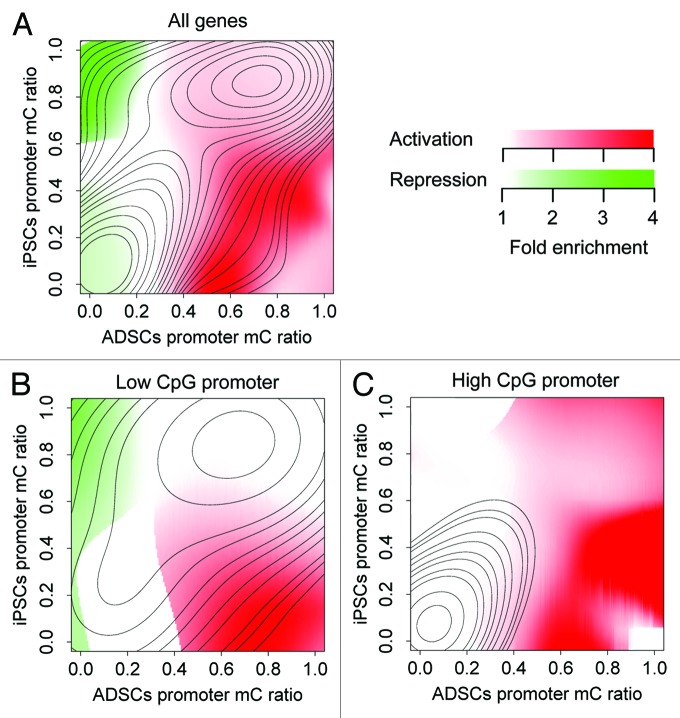
**Figure 3.** Differential methylation accompanied by differential expression in ADSCs and iPSCs. The distributions of activated and repressed genes are presented as enrichment relative to the background distribution of all genes. (**A**) All genes are plotted. Hypomethylation and hypermethylation were correlated with activation and repression, respectively. (**B**) Low-CpG promoters are plotted. Low-CpG promoters are responsible for the correlation of hypomethylation with activation and hypermethylation with repression. (**C**) High-CpG promoters are plotted. High-CpG promoters do not contribute to the correlation of hypermethylation with repression.

Because differential promoter methylation showed distinct patterns depending on the CpG content (Fig. 2A), we also investigated CpG content-related effects on the correlation with differential expression. Low- and high-CpG promoters exhibited different contributions to the correlation between methylation and expression. The low-CpG promoters were responsible for the correlation of hypomethylation with activation and the correlation of hypermethylation with repression (Fig. 3B; Fig. S2B). In contrast, few genes with high-CpG promoters were repressed (Fig. 3C; Fig. S2C). These results further indicate the complex nature of differential promoter methylation depending on CpG content.

Genes with differential promoter methylation showed enrichment in the unique set of gene ontology (GO) terms. Hypomethylated genes were enriched among genes related to developmental processes (Table S2), whereas hypermethylated genes were enriched among genes involved in cell-cell interactions and the immune response (Table S3). We also tested GO enrichment for genes whose promoter methylation was constantly high or low. Genes with consistently low methylation were enriched in housekeeping functions such as metabolic processes (Table S4). In contrast, genes with constantly high methylation were not significantly enriched in unique GO terms (Table S5).

We also analyzed the correlation between differential expression and differential methylation for the gene bodies. However, the correlation was subtle and seemed to be canceled out by heterogeneous effects from two classes of CpG content (Fig. S2D–F). Consequently, we concluded that differential methylation of promoters, rather than gene bodies, was correlated with differential expression and responsible for reprogramming to iPSCs.

### Methylation status of adipogenic master regulators

Although differential promoter methylation correlated well with differential gene expression for reprogramming to iPSCs (Fig. 3; Fig. S2), we detected only some differential methylation in promoters or gene bodies during differentiation to FatCs (Fig. 2B and D). To find epigenetic markers for fat differentiation in sites other than promoters and gene bodies, we took a closer look at known adipogenic regulators. During fat differentiation, key transcription factors such as PPARγ initiate adipogenesis by regulating an extensive network of genes that control lipid metabolism.^20^ Notably, several studies have documented that the binding ability of transcription factors may be affected by DNA methylation status in their target sites.^14^^,^^37^ Accordingly, we analyzed the methylation status of PPARγ-binding sites. For this purpose, we collected publicly available ChIP-Seq data for *PPARγ* ^7^ obtained in mature adipocytes (equivalent to FatCs in our study) and combined them with our MethylC-Seq and RNA-Seq data. We first analyzed the *C/EBPα* and *PPARγ* gene loci, as *C/EBPα* and *PPARγ* are known targets of *PPARγ*. In the *C/EBPα* locus, methylation of the PPARγ-binding regions was not altered between ADSCs and FatCs but was different between ADSCs and iPSCs (Fig. 4A), although *C/EBPα* expression was strongly upregulated in FatCs and not detectable in ADSCs (Fig. 4B; Fig. S6A). At the PPARγ locus, there were two types of PPARγ-binding regions that were differentially methylated from ADSCs to FatCs, or between iPSCs and ADSCs (Fig. 4C), whereas *PPARγ* expression was upregulated in FatCs but not in ADSCs (Fig. 4D; Fig. S6A). We next analyzed the methylation profiles of PPARγ-binding sites in FatCs (intended binding sites in iPSCs and ADSCs) on a genome-wide scale. As observed in the *C/EBPα* locus, methylation of PPARγ-binding sites was high in iPSCs and low in ADSCs and FatCs (Fig. 4, E–G). These results indicate that most intended binding sites were already hypomethylated in ADSCs even though PPARγ expression was not yet activated (Fig. 4D; Fig. S6A). Together with the methylation profile of promoters and gene bodies, these data suggest that epigenetic predetermination of the adipogenic fate in promoters, gene bodies, and PPARγ-binding sites is almost fully established in ADSCs.

**Figure:**
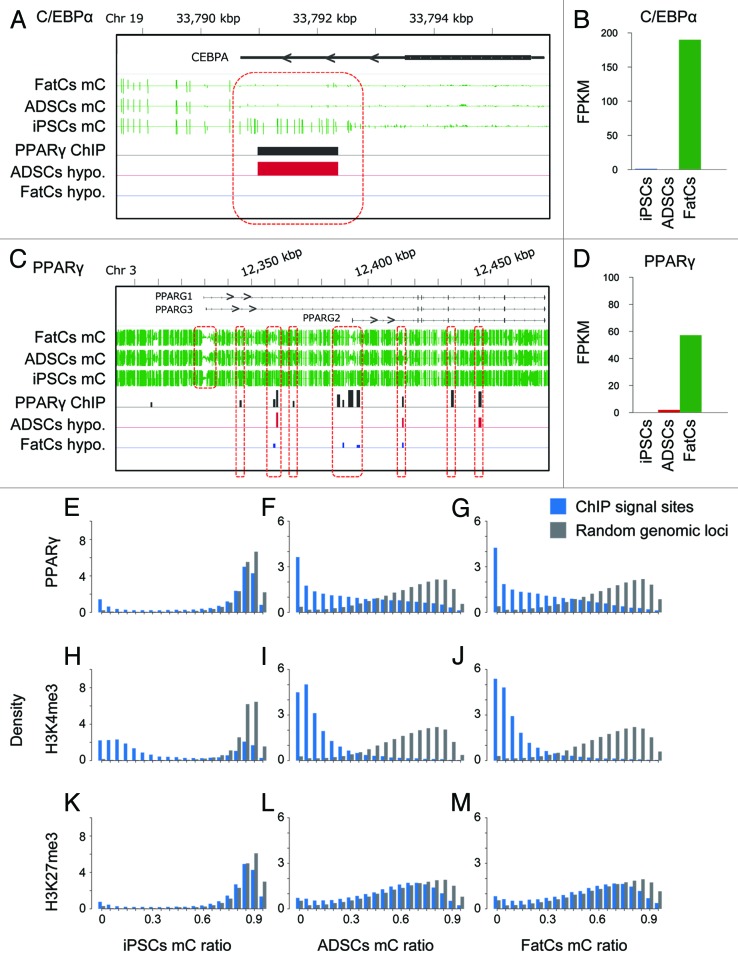
**Figure 4.** Methylation of PPARγ binding sites. (**A,C**) Shown are mC values in the *C/EBPα* (**A**) and *PPARγ* (**C**) gene loci. Black bars; PPARγ binding regions based on ChIP-Seq data, red bars; differentially methylated regions between ADSCs and iPSCs, blue bars; differentially methylated regions between ADSCs and FatCs. (**B, D**) The expression level of *C/EBPα* (**B**) and *PPARγ* (**D**) in each cell type. (**E-G**) The histogram of mC ratio at 52040 PPARγ sites compared with those for random genomic loci in each cell type. (**H-J**) The histogram for 33402 ChIP signal sites of H3K4me3 (activation mark) in each cell type. (**K-M**) The histogram for 54130 ChIP signal sites of H3K27me3 (repression mark) in each cell type.

We also analyzed ChIP-Seq data for several types of histone modification^7^ measured in FatCs, including H3K4me3, H3K4me2, H3K4me1, H3K27ac, H3K27me3, H3K36me3, and CTCF. Activation histone marks such as H3K4me3 and H3K27ac showed methylation profiles similar to those of PPARγ-binding sites; the intended binding sites of these histones were hypomethylated in ADSCs and FatCs, and methylated to a similar degree with random genomic loci in iPSCs (Fig. 4H–J; Fig. S3). In contrast, the binding sites of histones with repressive marks such as H3K27me3 consistently showed methylation as high as random genomic loci (Fig. 4K–M; Fig. S3). Thus, the methylation profile of PPARγ-binding sites resembled the profile of activation marks rather than that of repression marks, which is consistent with the role of PPARγ as a transcriptional activator.^21^

### Specific hypomethylation at PPARγ-binding sites is responsible for fat differentiation

Although most PPARγ-binding sites were already hypomethylated in ADSCs (Fig. 4E–G), several PPARγ-binding sites in the PPARγ gene locus were found to be later hypomethylated in differentiation to FatCs (Fig. 4C, blue bars). We analyzed the extent to which late hypomethylation in this timing was significant and specific to PPARγ-binding sites.

Significant hypomethylation during fat differentiation was detected at PPARγ-binding sites. Among 53 775 PPARγ-binding sites, 7826 were hypomethylated (Fig. 5A), showing a strong over-representation of hypomethylated binding sites (Fig. 5D). Remarkably, among the ChIP signal sites, the over-representation was the most significant for PPARγ-binding sites (Fig. S4), and not observed for H3K4me3- and H3K27me3-binding sites (Fig. 5B–D). Taken together with the stable methylation in promoters and gene bodies (Fig. 2B and D), these findings indicate unique hypomethylation at PPARγ-binding sites as a hallmark of differentiation to FatCs.

**Figure:**
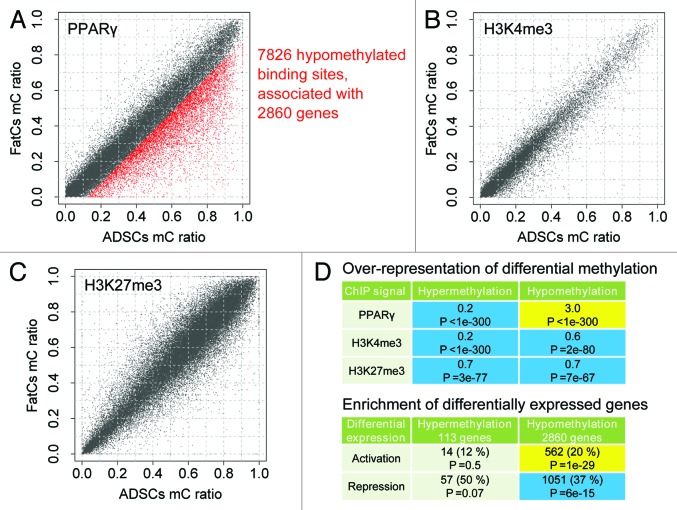
**Figure 5.** Differential methylation at PPARγ binding regions between ADSCs and FatCs. (**A-C**) Differential methylation of PPARγ binding sites (**A**), H3K4me3 (**B**), and H3K27me3 (**C**) between ADSCs and FatCs. Hypomethylated signal sites are over-represented specifically for PPARγ, but not for H3K4me3 or H3K27me3. The numbers of hypomethylated signal sites are 7826 out of 52040 for PPARγ, 971 out of 33402 for H3K4me3, and 1878 out of 54130 for H3K27me3. (**D**) Over-representation of differentially methylated signal sites in ADSCs and FatCs. Enrichment and *P* values were calculated by the binomial test. Over- and under-representation with *P* < 0.05 are colored in yellow and blue, respectively. (**E**) Enrichment of differentially methylated PPARγ binding sites among differentially expressed genes in ADSCs and FatCs. *P* values were calculated by the Fisher exact test. Enrichment and depletion (dis-enrichment) with *P* < 0.05 are colored in yellow and blue, respectively.

Hypomethylation at PPARγ-binding sites correlated well with the activation of target genes. Among 2,860 genes associated with hypomethylated PPARγ-binding sites, 562 were activated, showing a significant enrichment of activated genes (Fig. 5E). The activated genes were further tested for GO enrichment and found to be enriched in fat-related functions such as lipid metabolism (Table 1). Consequently, we determined these 562 genes as candidates of epigenetic markers for fat differentiation.

**Table:** **Table 1.** GO enrichment analysis of epigenetic markers for adipogenesis

Rank	Biological process			Molecular function			Cellular component		
	Term	N	P	Term	N	P	Term	N	P
1	small molecule metabolic process	143	6e-16	oxidoreductase activity	50	4e-6	cytoplasmic part	275	1e-9
2	lipid metabolic process	82	4e-13	oxidoreductase activity, acting on CH-OH group of donors	18	5e-5	mitochondrion	65	4e-5
3	cellular lipid metabolic process	70	3e-13	cofactor binding	25	1e-4	membrane	219	3e-5
4	carboxylic acid metabolic process	66	3e-11	oxidoreductase activity, acting on the CH-OH group of donors, NAD or NADP as acceptor	16	2e-4	microbody	15	3e-4
5	single-organism metabolic process	180	4e-10	coenzyme binding	19	7e-4	organelle outer membrane	16	5e-4
6	monocarboxylic acid metabolic process	43	5e-10	oxidoreductase activity, acting on the CH-CH group of donors	10	2e-3	peroxisome	13	4e-4
7	organic acid metabolic process	69	5e-10	catalytic activity	195	3e-3	outer membrane	16	5e-4
8	oxoacid metabolic process	68	6e-10	NAD binding	9	3e-3	endoplasmic reticulum	41	5e-4
9	single-organism process	366	6e-9	biotin carboxylase activity	4	4e-3	cytosol	108	1e-3
10	oxidation-reduction process	44	2e-8	ketosteroid monooxygenase activity	3	9e-3	mitochondrial membrane	31	1e-3

562 genes that exhibit a correlation with hypomethylation at PPARγ binding sites and activation show enrichment in fat-related functions. N: the number of query genes categorized with each term. P: *P* value adjusted for multiple testing with false discovery rate.

### Differentially methylated genes associated with PPARγ-binding sites are identified as novel epigenetic markers for fat differentiation

We next focused on highly confident candidate epigenetic markers whose hypomethylated PPARγ-binding sites were located in promoters and enhancers. We identified the adiponectin signaling axis as the most confident epigenetic marker. Adiponectin (ADIPOQ) is a protein hormone, or adipokine, which modulates a number of metabolic processes including glucose regulation and fatty acid catabolism. It has been reported that *ADIPOQ* is a PPARγ target gene possessing a PPARγ-binding site near its transcription start site (TSS).^22^ Furthermore, the pharmacological activation of PPARγ was found to induce *ADIPOQ* expression to regulate glucose metabolism in obesity.^23^ In the present study, we found three PPARγ-binding sites around the TSS of *ADIPOQ* that were hypomethylated during differentiation to FatCs (Fig. 6A; Fig. S6C). The hypomethylation was correlated with activation of *ADIPOQ* (Fig. 6B; Fig. S6). Notably, these binding sites were highly methylated in ADSCs and strictly hypomethylated in differentiation to FatCs. In addition to ADIPOQ, we found that the ADIPOQ receptor 2 gene (*ADIPOR2*) contained hypomethylated PPARγ-binding sites, one of which was located at the 30 kb upstream from the TSS (Fig. 6C). Hypomethylation at the distal binding site occurred during differentiation to FatCs and correlated with activation of *ADIPOR2* (Fig. 6D; Fig. S6A). Thus, the PPARγ-binding sites found in *ADIPOQ* and *ADIPOR2* exhibited a hypomethylation pattern distinct from the majority of other PPARγ-binding sites that were already hypomethylated in ADSCs. These results suggest that, in contrast to the epigenetic predetermination observed in the locus of the adipogenic master regulator gene, *C/EBPα*, the PPARγ−binding sites of late-expressing genes such as *ADIPOQ* and *ADIPOR2* are differentially methylated after induction of adipogenesis, thus providing markers for later-stage epigenetic regulation of adipose differentiation.

**Figure:**
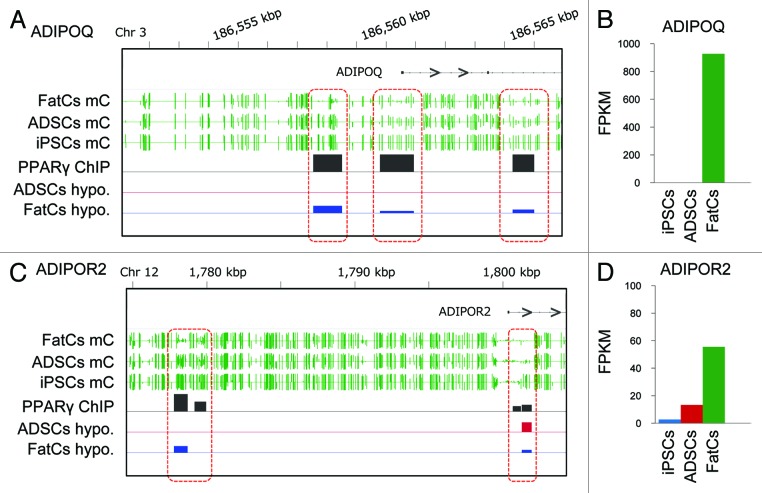
**Figure 6.** Epigenetic markers for adipogenesis. (**A,C**) mC values in the *ADIPOQ* (**A**) and *ADIPOR2* (**C**) gene loci for each cell type. (**B,D**) The expression levels of *ADIPOQ* (**B**) and *ADIPOR2* (**D**) in each cell type.

One drawback of MethylC-Seq is that the method cannot distinguish methylation and hydroxymethylation. Thus, we measured not only methylation ratios but also hydroxymethylation ratios for epigenetic markers in *ADIPOQ* (Materials and Methods). The results showed that the hydroxymathylation ratios were consistently low in ADSCs and FatCs (Fig. S6C), suggesting that the contribution of hydroxymathylation was not large in these loci.

## Discussion

The findings of our study suggest a model of adipogenic differentiation involving two modes of epigenetic mechanisms. In the first mode, promoters, gene bodies, and the majority of PPARγ−binding sites are hypomethylated in ADSCs before adipogenic induction. In the second, a subset of PPARγ−binding sites in late-expressing genes such as *ADIPOQ* and *ADIPOR2* is hypomethylated during adipogenesis.

DNA methylation has been traditionally shown to contribute to the regulation of gene expression and determination of cell identity.^10^^,^^19^ However, our study using MethylC-Seq and RNA-Seq showed the unexpected result that methylation in promoters and gene bodies was not extensively changed in differentiation from ADSCs to FatCs, which contrasts with the reprogramming of ADSCs to iPSCs, even though the gene expression variation in differentiation and reprogramming were similar. These data suggest that promoters and gene bodies of ADSCs are already epigenetically primed for the mature FatC lineage prior to the expression of FatC-specific genes.

In addition to methylation in promoters and gene bodies, we also observed that the methylation status of PPARγ-binding sites was very similar between ADSCs and FatCs on a genome-wide scale. This result is somewhat surprising because we first expected that PPARγ-binding sites are hypomethylated after induction of adipogenic differentiation. Given that the DNA methylation status of the target sequence has been shown to affect transcription factor binding and the associated gene expression,^13^^,^^14^ it is likely that hypomethylation of PPARγ-binding sites is accompanied by PPARγ binding and target gene expression. However, our data indicate that most PPARγ-binding sites are already hypomethylated prior to PPARγ activation. Consistent with this notion, a previous study using PPARγ ChIP-Seq and DNase I hypersensitive site analysis revealed that many PPARγ−binding sites develop an open chromatin structure shortly after induction of adipocyte differentiation in 3T3-L1 cells prior to PPARγ activation.^15^^,^^24^ Similarly, Mikkelsen et al. showed that ~77% of all PPARγ-binding sites detected in adipocytes are matched to the H3K4me and H3K27ac region in preadipocytes.^7^ Taken together, these data demonstrate that the intended PPARγ−binding sites in ADSCs and preadipocytes already gain an “open” state as mature FatCs with regard to both DNA methylation and histone modification, even before the initiation of adipogenesis, in a pre-set methylation profile.

In contrast to the pre-set methylation state at the majority of PPARγ−binding sites, closer inspection using our MethylC-Seq and ChIP-Seq data identified differentially methylated regions at a subset of PPARγ-binding sites during adipogenesis. One of the genes containing differentially methylated regions is *ADIPOQ*, which is expressed at the terminal differentiation stage of adipogenesis and regulates metabolic processes as a protein hormone. In addition to *ADIPOQ*, *ADIPOQ receptor 2* gene (ADIPOR2) also contained differentially methylated regions. These data suggest that late-expressing genes such as *ADIPOQ* and *ADIPOR2* are differentially methylated during adipogenesis, whereas early genes such as C/EBPα are already hypomethylated before induction of adipogenesis.

To gain further insight into the epigenetic transitions at the selected DMRs, we measured H3K4me3, H3K27me3, and H3K27ac histone marks for iPSCs, ADSCs, and FatCs (Fig. S6B). Interestingly, we found that histone marks of these DMRs are not associated with DNA methylation state in ADSCs. In the C/EBPα locus, ADSCs exhibited a complex state where the activating mark H3K4me3 was not enriched, and the repressive mark H3K27me3 was enriched in spite of DNA hypomethylation, implying that DNA hypomethylation occurs first but repressive histone mark still maintain the transcription silence of the gene. Furthermore, the ADIPOQ and ADIPOR2 loci exhibited a different but also complex state that the activating mark H3K4me3 at DMR1 was more enriched in ADSCs than in FatCs although the DMR1 is methylated in ADSCs and hypomethylated in FatCs, whereas the enrichment of the activating mark H3K4me3 was not observed at DMR2 or DMR3 in ADSCs, implying that the relationship between the genomic context (outside the genes or intergenic) and histone mark. Together, we speculate that the selected DMRs in ADSCs might represent “bivalent” domains that harbor both activating and repressive chromatin signatures for timely activation of developmental genes upon differentiation cues.^38^ Further analysis is needed to address whether the bivalent chromatin state are also observed in other locus, and if so, how the bivalent state affects gene expression in ADSCs.

Recently, methylation analysis based on Illumina’s Infinium technology, HumanMethylation BeadChip, was used to map the DNA methylation state on a genome-wide scale.^25^^,^^26^ Some studies have shown that the BeadChip analysis is a useful and reliable approach to detect disease-related and age-related methylation change.^27^^,^^28^ However, by comparing the mC patterns detected in our MethylC-Seq analysis with CpG sites targeted by Illumina Infinium HumanMethylation450K BeadChip probes, we observed that the differentially methylated regions in *ADIPOQ* and *ADIPOR2* loci could not be detected by BeadChip analysis (Fig. S5). This finding suggests that BeadChip analysis is not sufficient for comprehensive studies that aim to understand genomic global trends and detect novel differentially methylated regions.

Our MethylC-Seq analysis clearly shows the unique state of somatic stem cells in which the intended binding sites for master regulator genes are already hypomethylated before induction of differentiation. To explore the possibility that epigenetic predetermination occurs in other somatic stem cells, comprehensive analyses using DNA methylation, mRNA expression, and ChIP for master regulators, as well as hydroxymethylation, in other differentiation models are necessary in future studies.

## Materials and Methods

### Cell culture

ADS cells obtained from Invitrogen (R7788110), and ADS-derived iPS cells^18^ were cultured under the recommended conditions as previously described.^29^ For the in vitro differentiation of ADS cells to mature adipocytes, ADS cells were plated on 10-cm^2^ dishes with growth media. Differentiation was induced for 14 d using medium consisting of Dulbecco’s modified Eagle medium/nutrient mixture F12 (DMEM/F12), 10% knockout serum replacement (KSR), and an adipogenic cocktail (0.5 mM IBMX, 0.25 mM dexamethasone, 1 mg/ml insulin, 0.2 mM indomethacin, and 1 mM pioglitazone). For collection of mature adipocytes, the cells were detached with trypsin and then neutralized. After the detached cells were centrifuged, floating cells were transferred into new tubes.

### Library generation for directional RNA-Seq and MethylC-Seq

Total RNA was isolated from cell pellets using an RNeasy mini kit (Qiagen) and treated with DNase I (Qiagen) for 30 min at room temperature. After ethanol precipitation, rRNA (rRNA) was depleted from 5 μg of total RNA by biotinylated LNA oligonucleotide probes complementary to 5S, 5.8S, 12S, 18S, and 28S rRNA using RiboMinus (Life Technologies) according to the manufacturer’s instructions. Purified RNA (50 ng) was fragmented by metal hydrolysis in a 1 × fragmentation buffer (Life Technologies) for 15  min at 70 °C, and the reaction was stopped by the addition of 2 µl of fragmentation stop solution (Life Technologies). Fragmented RNA was used to generate strand-specific RNA-Seq libraries according to the Directional mRNA-Seq Library Preparation Protocol (Illumina). MethylC-Seq libraries were generated by bisulfite-based methods as previously described.^18^ Briefly, 5 μg of genomic DNA was fragmented to 100–400 bp followed by adaptor ligation and size selection to 275–375 bp (150–250 bp insert). Adaptor-ligated DNA was bisulfite-converted and amplified by 8 (ADSCs) or 6 (iPSCs and FatCs) cycles of PCR.

### High-throughput sequencing and data analysis

RNA-Seq and MethylC-Seq libraries were prepared from two biological replicates (two different individuals of Caucasian female) for each experimental condition, and sequenced up to 75 cycles using the Illumina Genome Analyzer IIx or HiSeq2000. Image analysis and base calling were performed using the standard Illumina pipeline; a control library sequenced in a single lane of each flow cell was used for matrix and phasing calculations. For RNA-Seq, the total numbers of sequenced reads were 416, 309, and 393 million for ADSCs, iPSCs, and FatCs, respectively. For MethylC-Seq, the total numbers of sequenced reads were 2396, 2645, and 2449 million for ADSCs, iPSCs, and FatCs, respectively. Among 26 million captured CpG sites, we obtained the average coverage of 28X for ADSCs, 36X for iPSCs, and 28X for FatCs. All data were submitted to the Sequence Read Archive (SRP003529).

RNA-Seq reads were mapped to the reference by the TopHat program,^30^ which can perform spliced alignment. Gene expression was measured as fragments per kilobase of transcript per million mapped reads (FPKM) computed by the Cufflinks program.^31^ Differential gene expression was evaluated according to the fold change in FPKM. Activated and repressed genes were determined according to the threshold of a 2-fold FPKM change.

MethylC-Seq reads were mapped by the Bisulfighter program,^32^ which has been recently confirmed to be accurate for bisulfite-converted DNA alignment. For each CpG in the reference, methylation was measured by the mC value, i.e., the fraction of non-converted (C-C matching) reads relative to the total reads mapped at the CpG. Then, for each region of interest (a promoter or a ChIP signal site), the mC ratio was computed as the average of mC values over all CpGs in the region. To compare methylation to the genomic background (Fig. 4, E–M; Fig. S4), mC ratios were also calculated for random genomic loci whose length distribution was the same as the original regions. Differential methylation was evaluated by the difference in mC ratio. Hypomethylated and hypermethylated regions were determined by the threshold of the fifth percentile of mC ratio differences calculated from random genomic loci.

Publicly available ChIP-Seq data for mature adipocytes by Mikkelsen et al.^7^ were obtained from the Sequence Read Archive (SRP002343). Mature adipocytes used by Mikkelsen et al. are basically equivalent to FatCs used in our study. Both studies used adipose derived stem cells isolated from lipoaspirate tissue whose race and gender backgrounds were concordant (Caucasian female). Differentiation was induced by the same protocol between the two studies. Based on these facts, we performed integrated analysis of ChIP-Seq data with our MethylC-Seq and RNA-Seq data.

ChIP-Seq reads were aligned by the Bowtie program,^33^ and signal sites were detected using the MACS program,^34^ for which mapping results for the whole cell extract sample were used as a control. Signal sites were associated with genes if they were located within 3,000 bp. The differentially methylated signal sites were counted and tested for over- or under-representation in the differentially expressed genes (Fig. 5A and D) by the binomial test with an occurrence probability of 5%.

The reference sequence and annotations for the human genome were taken from UCSC hg19. Promoters were defined as regions between –1000 bp and +500 bp around the TSS, whereas gene bodies were defined as regions between +2000 bp from the TSS and the corresponding transcription end sites. Genes were excluded from the analysis if their loci were too short to place the TSS or gene-body window. Genome browser snapshots were produced by the IGV program.^35^ Gene ontology (GO) enrichment analysis was performed by the GOrilla tool.^36^

### RT-qPCR

Samples were analyzed by qPCR, using SYBR Green dye (TOYOBO). Samples were run in triplicate and expression was normalized to the levels of the housekeeping controls, universal 36b4 for human. Error bars are mean ± standard error.

### ChIP-qPCR at selected loci

10^7^ cells are crosslinked with 1% formaldehyde for 10 min at room temperature. After quenching with 0.125 M glycine, the cell pellet are resuspended in lysis buffer (50 mM Hepes, 140 mM NaCl, 1 mM EDTA, 10% glycerol, 0.5% NP-40, 0.25% TX-100, and proteinase inhibitors) for 10 min. After spin down the pellets are further resuspended in 10 mM Tris, 200 mM NaCl, 1 mM EDTA, 0.5 mM EGTA. Finally the pellets are resuspened in 10 mM Tris, 100 mM NaCl, 1 mM EDTA, 0.5 mM EGTA, 0.1% Na-Deoxycholate and 0.5% N-lauroylsarcosine. Sonication was performed using Bioruptor (Diagenode) (30 s on, 30 s off, total 15 min). The sonicated chromatin was then aliqoted and incubated with 2 μg of antibodies overnight, followed by washing, de-crosslinking and DNA precipitation. The antibodies used for ChIP include anti-H3K4me3 (Abcam, ab8580), anti-H3K27me3 (Active Motif, 39155), and anti-H3K27ac (Active Motif, 39133). Immunoprecipitated DNA was analyzed by qPCR using multiple primers specific to each DMR. The primers used for qPCR are listed in Table S6.

### Methylation and hydroxymethylation assay

For selected DMRs between ADSCs and FatCs, we measured not only methylated cytosines but also hydroxymethylated cytosines using EpiMark kit (New England BioLabs). The method is a kind of (hydroxy) methylation-sensitive restriction enzyme and qPCR assay where hydroxymethylated sites are protected by glycosylation, and methylated sites are specifically cleaved by MspI, while unmethylated sites are specifically cleaved by HpaII. Methylation and hydroxymethylation ratios can be calculated from qPCR values for digestion products and uncut controls. All protocols were following the manufacturer's instruction. Since the restriction enzymes target CCGG sequences, DMRs not containing the motif were excluded from the analysis. The qPCR primers were 5′-CTCCCAAAAT GCTGGGATTA CA-3′ and 5′-GTTGATGTAT GTGCTTCAGG GTAGTT-3′ for DMR1 targeting the CCGG site at chr3:186558290, and 5′-CCGAAGCCCA AGCTGGGTTG TA-3′ and 5′-ACAATTGTCA TTTCCCATTG GCC-3′ for DMR2 targeting the CCGG site at chr3:186560385.

## Supplementary Material

Additional material

## Notes

10.4161/epi.29856
