# Supplementary material for: Methylome, transcriptome, and PPARγ cistrome analyses reveal two epigenetic transitions in fat cells
Source: Epigenetics. 2014 Jul 10;9(9):1195–206. doi: 10.4161/epi.29856 (PMC4169011; doi:10.4161/epi.29856)
Supplement: Additional material [file epi-9-1195-s01.pdf]

Landes Bioscience

www.landesbioscience.com

Supplemental Material to:

Hitomi Takada, Yutaka Saito, Toutai Mituyama, Zong Wei, Eiji Yoshihara, Sandra Jacinto, Michael Downes, Ronald M Evans, and Yasuyuki S Kida

Methylome, transcriptome, and PPARγ cistrome analyses reveal two epigenetic transitions in fat cells

Epigenetics 2014; 9(9)
http://dx.doi.org/10.4161/epi.29856

http://www.landesbioscience.com/journals/epigenetics/article/29856/

Supplemental Figure 1

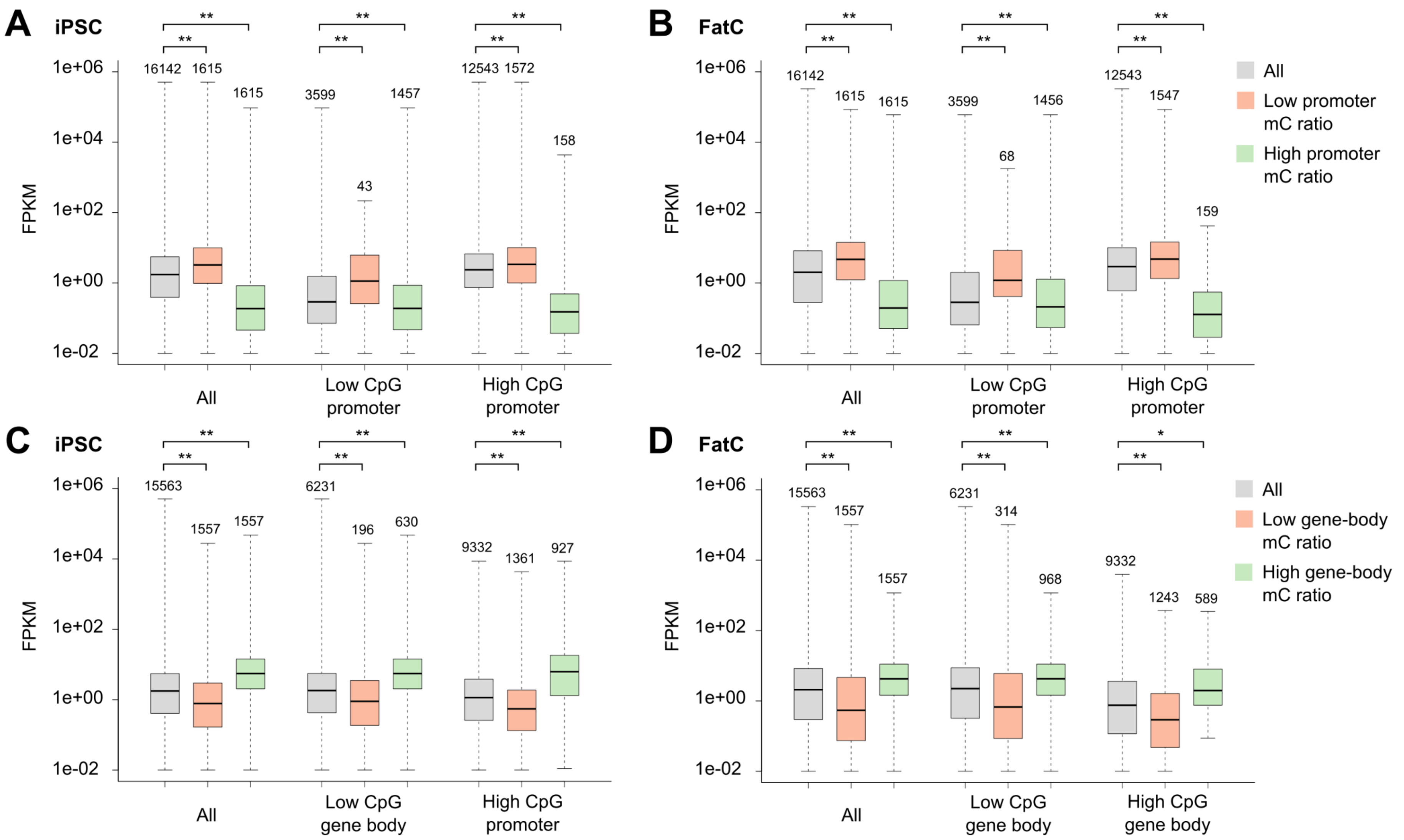

Supplemental Fig.1. Relationship between promoter or gene-body methylation and gene expression in iPSCs and FatCs

(A,B) Analysis similar to that described for Figure 1B was performed for the mC ratios calculated from promoters in iPSCs (A) and FatCs (B)

(C,D) Analysis similar to that described for Figure 1D was performed for the mC ratios calculated from gene bodies in iPSCs (C) and FatCs (D). Symbols *: P<0.05; **: P<10-3. Box: 25-75th percentile.

Supplemental Figure 2

Differential promoter methylation

A All genes

Differential expression	Hypermethylation 291 genes	Hypomethylation 819 genes
Activation	37 (13 %) *P*=0.1	248 (30 %) *P*=3e-29
Repression	174 (60 %) *P*=0.01	332 (41 %) *P*=4e-14

B Genes with low CpG promoters

Differential expression	Hypermethylation 245 genes	Hypomethylation 258 genes
Activation	30 (12 %) *P*=0.1	86 (33 %) *P*=2e-13
Repression	151 (62 %) *P*=5e-3	92 (36 %) *P*=7e-9

C Genes with high CpG promoters

Differential expression	Hypermethylation 46 genes	Hypomethylation 561 genes
Activation	7 (15 %) *P*=0.6	162 (29 %) *P*=4e-17
Repression	23 (50 %) *P*=0.4	240 (43 %) *P*=2e-7

Differential gene-body methylation

D All genes

Differential expression	Hypermethylation 190 genes	Hypomethylation 570 genes
Activation	33 (17 %) *P*=0.2	86 (15 %) *P*=0.5
Repression	107 (58 %) *P*=0.2	312 (55 %) *P*=0.3

E Genes with low CpG gene bodies

Differential expression	Hypermethylation 67 genes	Hypomethylation 181 genes
Activation	10 (15 %) *P*=0.5	10 (6 %) *P*=5e-5
Repression	33 (49 %) *P*=0.3	106 (59 %) *P*=0.1

F Genes with high CpG gene bodies

Differential expression	Hypermethylation 123 genes	Hypomethylation 389 genes
Activation	23 (19 %) *P*=0.2	76 (20 %) *P*=0.01
Repression	74 (60 %) *P*=0.08	206 (53 %) *P*=0.4

Supplemental Fig. 2. Enrichment of differentially methylated genes among differentially expressed genes in ADSCs and iPSCs

(A-C) Differential promoter methylation of all genes (A), genes with low-CpG promoters (B), and genes with high-CpG promoters (C). (D-F) Differential gene-body methylation of all genes (D), low-CpG gene bodies (E), and high-CpG gene bodies (F). *P*-values were calculated by the Fisher exact test. Enrichment and depletion (dis-enrichment) with $P < 0.05$ are colored in yellow and blue, respectively.

Supplemental Figure 3

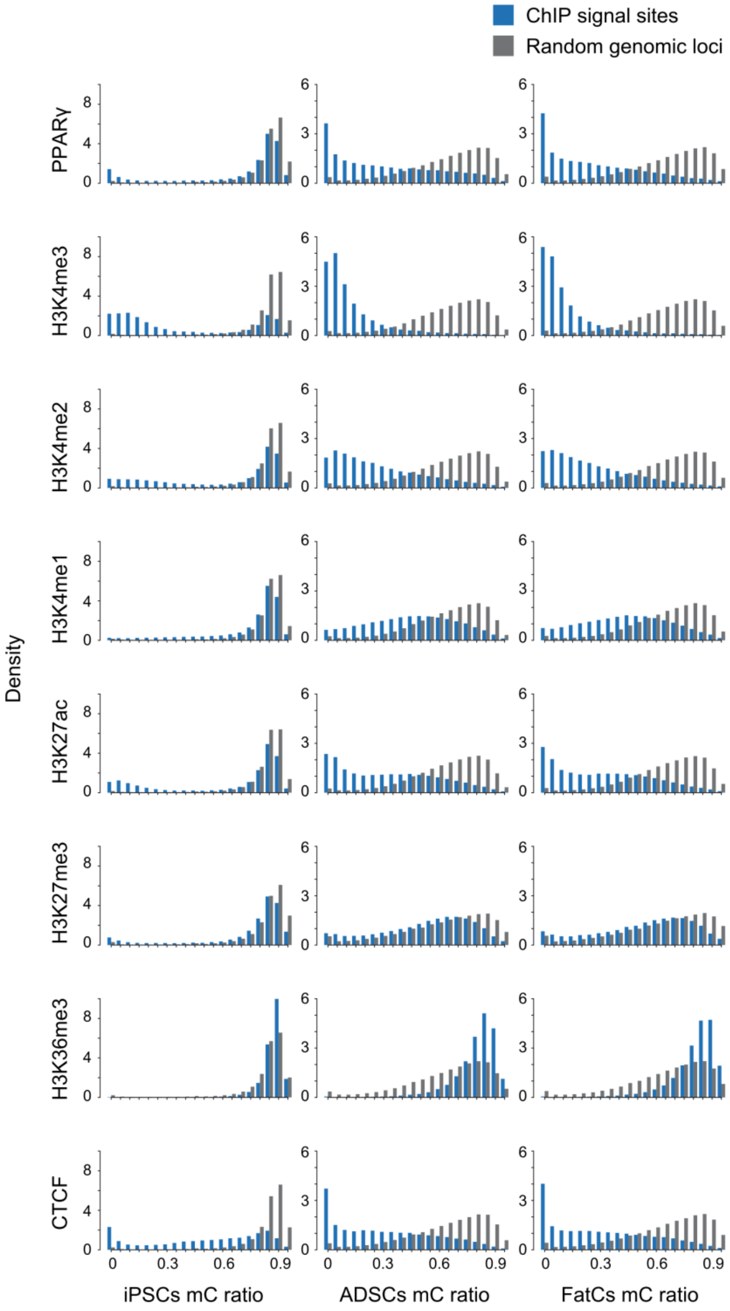

Supplemental Fig. 3. Methylation of 8 types of ChIP signal sites

The histogram of mC ratio at ChIP signal sites for PPARγ, H3K4me3, H3K4me2, H3K4me1, H3K27ac, H3K27 me3, H3K36me3, and CTCF are presented, and are compared to random genomic loci.

Supplemental Figure 4

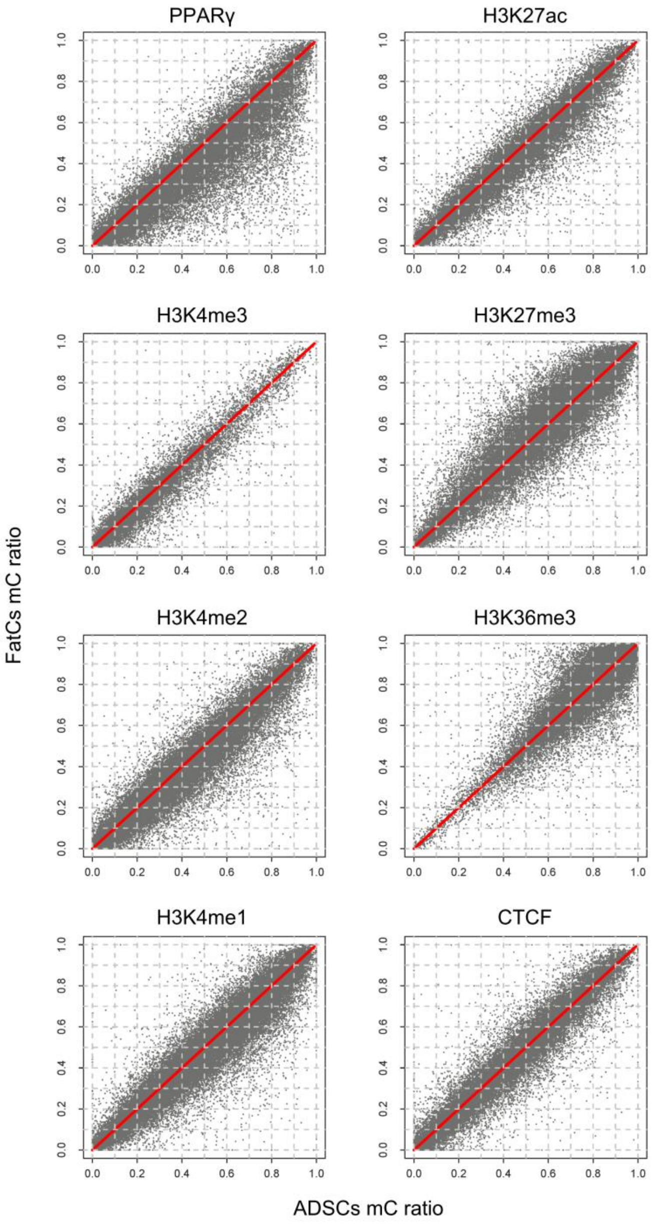

Supplemental Fig. 4. Differential methylation of 8 types of ChIP signal sites in ADSCs and FatCs

Hypomethylated signal sites are over-represented specifically for PPARγ but not for other ChIP signal sites.

Supplemental Figure 5

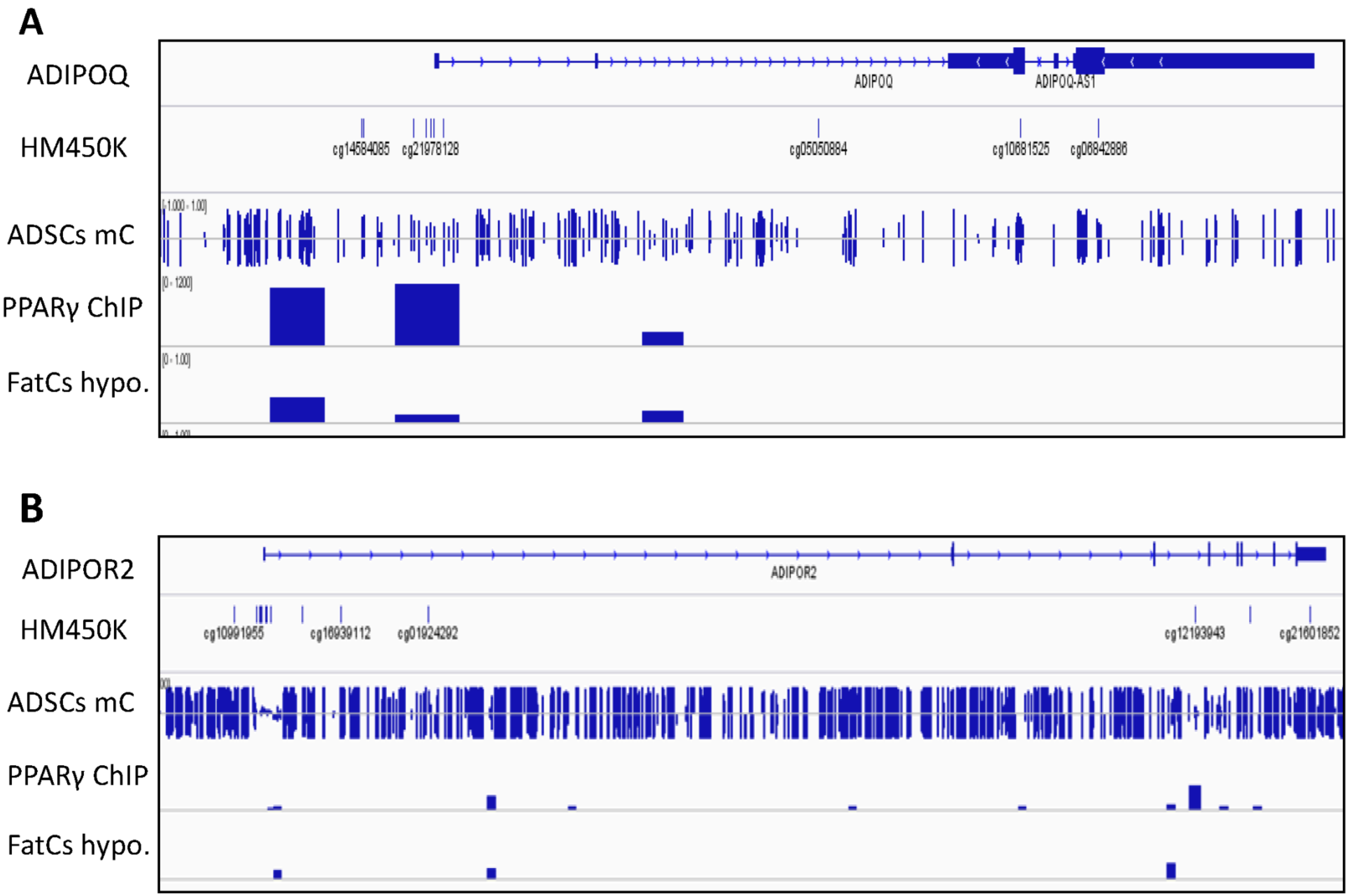

Supplemental Fig. 5. Comparison of methylated CpG sites detected in our MethylC-Seq analysis to CpG cites in Illumina HumanMethylation450K Bead Chip probes

(A,B) The positions of methylated CpGs in *ADIPOQ* (A) and *ADIPOR2* (B) gene loci. HM450K CpG sites in HumanMethlation450K BeadChip probes; ADSCs mC, methylated CpGs detected in our MethylC-Seq data for ADSCs; PPARγ ChIP, PPARγ-binding sites in FatCs; FatCs hypo, PPARγ-binding sites hypomethylated during differentiation of ADSCs to FatCs.

Supplemental Figure 6

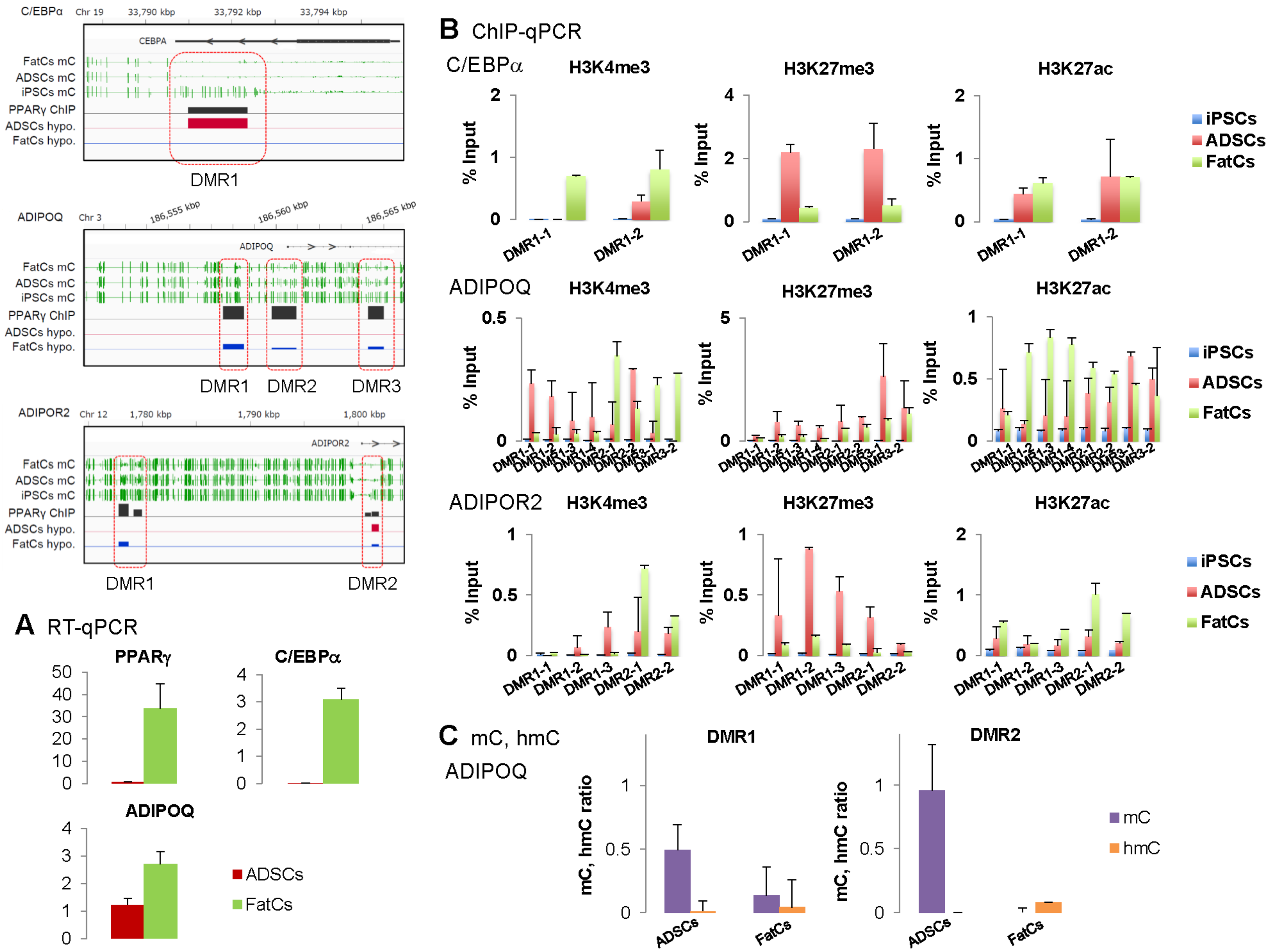

Supplemental Fig. 6. Gene expression, histone modification, methylation and hydroxymethylation at selected loci measured by conventional methods
(A) Gene expression measured by RT-qPCRT. (B) H3K4me3, H3K27me3 and H3K27ac histone marks measured by ChIP-qPCR. The legends such as DMR1-1 and 1-2 represent different primer pairs used for the same DMR. (C) Ratios of methylated cytosine (mC) and hydroxymethylated cytosine (hmC) measured by restriction enzyme and qPCR assay. Top left, illustrations of selected loci.

Supplemental Table 1. GO enrichment analysis of expression markers for FatCs

Rank	Biological process			Molecular function			Cellular component		
	Term	N	P	Term	N	P	Term	N	P
1	small molecule metabolic process	145	1e-51	oxidoreductase activity	54	1e-19	cytoplasmic part	210	9e-33
2	lipid metabolic process	100	3e-48	cofactor binding	32	1e-16	mitochondrion	74	7e-24
3	cellular lipid metabolic process	89	2e-47	catalytic activity	151	3e-15	lipid particle	17	9e-16
4	single-organism metabolic process	163	3e-38	oxidoreductase activity, acting on CH-OH group of donors	21	9e-13	mitochondrial part	45	1e-11
5	carboxylic acid metabolic process	75	5e-34	oxidoreductase activity, acting on the CH-OH group of donors, NAD or NADP as acceptor	20	1e-12	microbody	18	5e-11
6	oxoacid metabolic process	76	4e-31	coenzyme binding	23	6e-12	intracellular part	243	1e-10
7	monocarboxylic acid metabolic process	54	7e-31	lyase activity	19	3e-9	cytosol	85	3e-10
8	organic acid metabolic process	76	9e-31	oxidoreductase activity, acting on the CH-CH group of donors	12	2e-8	organelle membrane	82	3e-10
9	fatty acid metabolic process	43	8e-28	transferase activity, transferring acyl groups	21	4e-8	organelle lumen	36	2e-9
10	single-organism biosynthetic process	76	3e-27	organic acid binding	18	4e-7	peroxisomal membrane	12	3e-9

GO enrichment analysis was performed on 314 expression markers for FatCs, which were defined by the following criteria: FPKM is more than twice as large in FatCs as in the other cell types; FPKM in FatCs is in the top 10% of all genes. N: the number of query genes categorized with each term. P: P-value adjusted for multiple testing with false discovery rate.

Supplemental Table 2. GO enrichment analysis of genes with promoter hypomethylation in iPSCs relative to ADSCs

Rank	Biological process			Molecular function		
	Term	N	P	Term	N	P
1	embryonic morphogenesis	39	3e-4	nucleic acid binding transcription factor activity	65	4e-2
2	anatomical structure morphogenesis	83	5e-4	Rho guanyl-nucleotide exchange factor activity	12	4e-2
3	skeletal system morphogenesis	16	5e-3	Ras guanyl-nucleotide exchange factor activity	15	5e-2
4	embryonic organ morphogenesis	17	9e-3			
5	developmental process	206	0.01			
6	anatomical structure development	135	0.02			
7	organ morphogenesis	33	0.04			
8	negative regulation of gene expression	62	0.04			
9	negative regulation of RNA metabolic process	60	0.04			
10	embryonic skeletal system morphogenesis	12	0.04			

GO enrichment analysis was performed on 819 genes whose promoters were hypomethylated from ADSCs to iPSCs. N: the number of query genes categorized with each term. P: P-value adjusted for multiple testing with false discovery rate.

Supplemental Table 3. GO enrichment analysis of genes with promoter hypermethylation in iPSCs relative to ADSCs

Rank	Biological process			Cellular component		
	Term	N	P	Term	N	P
1	cell adhesion	33	2e-5	extracellular region part	39	3e-5
2	biological adhesion	33	1e-5	extracellular matrix	16	1e-3
3	homophilic cell adhesion	12	6e-4	extracellular space	28	2e-3
4	defense response	35	2e-3	extracellular region	37	9e-3
5	defense response to virus	11	5e-3	interstitial matrix	4	9e-3
6	immune system process	42	5e-3	plasma membrane	68	2e-2
7	immune response	30	6e-3			
8	cell-cell adhesion	17	7e-3			
9	defense response to other organism	15	7e-3			
10	regulation of inflammatory response	13	8e-3			

GO enrichment analysis was performed on 291 genes whose promoters were hypermethylated from ADSCs to iPSCs. N: the number of query genes categorized with each term. P: P-value adjusted for multiple testing with false discovery rate.

Supplemental Table 4. GO enrichment analysis of genes with constantly low promoter methylation

Rank	Biological process			Molecular function			Cellular component		
	Term	N	P	Term	N	P	Term	N	P
1	cellular macromolecule metabolic process	731	4e-26	protein binding	909	6e-15	intracellular part	1351	1e-31
2	macromolecule metabolic process	768	2e-19	nucleic acid binding	429	3e-13	intracellular organelle part	762	2e-17
3	cellular metabolic process	888	2e-18	binding	1249	5e-13	intracellular organelle	946	1e-16
4	primary metabolic process	891	6e-17	heterocyclic compound binding	601	9e-10	nuclear part	350	2e-16
5	nucleic acid metabolic process	472	7e-16	organic cyclic compound binding	606	2e-9	organelle part	769	8e-16
6	organic substance metabolic process	908	1e-15	DNA binding	299	1e-7	organelle	953	1e-15
7	nucleobase-containing compound metabolic process	527	2e-15	RNA binding	136	9e-7	nucleus	604	1e-15
8	heterocycle metabolic process	538	5e-14	sequence-specific DNA binding transcription factor activity	150	9e-7	intracellular membrane-bounded organelle	844	4e-15
9	metabolic process	943	8e-14	nucleic acid binding transcription factor activity	150	9e-7	macromolecular complex	529	6e-15
10	gene expression	131	1e-13	regulatory region DNA binding	62	3e-3	membrane-bounded organelle	857	3e-14

The threshold for mC ratio was set to 0.037 so that the lowest 10 percent (2010 genes) was selected. N: the number of query genes categorized with each term. P: P-value adjusted for multiple testing with false discovery rate.

Supplemental Table 5. Genes with constantly high promoter methylation.

Rank	Biological process			Molecular function			Cellular component		
	Term	N	P	Term	N	P	Term	N	P
1	fertilization	21	0.01	receptor activity	183	8e-6	extracellular region	199	2e-10
2	sensory perception of taste	13	0.02	transmembrane signaling receptor activity	152	1e-5	intermediate filament	48	6e-10
3	multicellular organismal development	61	0.02	signaling receptor activity	163	1e-5	keratin filament	27	2e-6
4	protein activation cascade	17	0.03	G-protein coupled receptor activity	111	4e-5	extracellular region part	150	1e-5
5	detection of stimulus	44	0.03	molecular transducer activity	186	2e-4	extracellular space	113	8e-5
6				signal transducer activity	186	3e-4	plasma membrane	349	3e-3
7				structural constituent of epidermis	6	1e-3	integral component of membrane	460	0.03
8				structural constituent of cytoskeleton	22	2e-3			
9				structural molecule activity	80	4e-3			
10				cytokine activity	36	8e-3			

The threshold for mC ratio was set to 0.70 so that the highest 10 percent (2123 genes) was selected. N: the number of query genes categorized with each term. P: P-value adjusted for multiple testing with false discovery rate.

Supplemental Table 6. Primers used for ChIP-qPCR analysis

Gene	Primer name	Forward	Reverse
C/EBPa	DMR1-1	5'- AAAGCTGAGGGCAAAGGAGAAC-3'	5'- TCGGATACTTGCCAAAATGAGAC-3'
	DMR1-2	5'- ATAAAATGGTGGTTTAGCAGAGACG-3'	5'-TGCTTTATCAGCCGATATCAACACT-3'
ADIPOQ	DMR1-1	5'-CTTGGTGTGATTGTGGGGCTAAA-3'	5'-AACAGAGCGAGAATCCATCTCAAAA-3'
	DMR1-2	5'-GCTTTCACAATGTCACTGACTGA-3'	5'-TCTTCTAACTTTATAAGAATGCAGCTGG-3'
	DMR1-3	5'-AACTACCCTGAAGCACATACATCAACC-3'	5'-GGCACAAGTCTAGGATTTGAACCA-3'
	DMR1-4	5'-TTATGCCAGCCCTGAAACTACC-3'	5'-CCCTATGCTTTCTAATTCCTCATGC-3'
	DMR2-1	5'-CCATCTCCTCCTCACTTCCATTCT-3'	5'-GGTGATCCCAGCCAGAGGCTAT-3'
	DMR2-2	5'-TTGTTTTCCTGCTGTGGTTTTG-3'	5'-GAGCTTCAGTCAAAGCACTTGTGT-3'
	DMR3-1	5'-GACCCTGTCCTTGCATTTCACA-3'	5'-GGGCCATACTGTGATCATTTATCTTC-3'
	DMR3-2	5'-TGACCACGATACTTTTCTCCTCCT-3'	5'-CTTTCCAAGATCCCAGAGCTACAC-3'
ADIPOR2	DMR1-1	5'-CACAAAAGAAACCTTTACCCAATCC-3'	5'-GCTCGCTCTCACGTATTATGACTCT-3'
	DMR1-2	5'-CAGTACCCTTGCTGAATTTCTTTCC-3'	5'-ATAGGACTCCCTCTGCCCCAGT-3'
	DMR1-3	5'-CTTCGACCTCCAGACAGACACA-3'	5'-GTCGCTTGGATTGGGTAAAGGT-3'
	DMR2-1	5'-AAAGCATCCATCTACCCATAGCA-3'	5'-ACATAAGGGTGGCCTTTGATAAGA-3'
	DMR2-2	5'-TCTGATTGTAACTGTCTGTCCTCCA-3'	5'-TTTTAGGCTCTGGAAATCCTACTCC-3'
